# Preserving Posterior Complex Can Prevent Adjacent Segment Disease following Posterior Lumbar Interbody Fusion Surgeries: A Finite Element Analysis

**DOI:** 10.1371/journal.pone.0166452

**Published:** 2016-11-21

**Authors:** Yun-Peng Huang, Cheng-Fei Du, Cheng-Kung Cheng, Zheng-Cheng Zhong, Xuan-Wei Chen, Gui Wu, Zhe-Cheng Li, Jin-Duo Ye, Jian-Hua Lin, Li Zhen Wang

**Affiliations:** 1 Department of Orthopaedics, The First Affiliated Hospital of Fujian Medical University, Fuzhou City, Fujian Province, 350005, China; 2 Tianjin Key Laboratory for Advanced Mechatronic System Design and Intelligent Control, Tianjin University of Technology, Tianjin, 300384, China; 3 Key Laboratory for Biomechanics and Mechanobiology of Ministry of Education, International Research Center for Implantable and Interventional Medical Devices, School of Biological Science and Medical Engineering, Beihang University, Beijing, 100191, China; 4 Orthopaedic Device Research Center, National Yang-Ming University, 11221, Taipei, China; Emory University School of Medicine, UNITED STATES

## Abstract

**Objective:**

To investigate the biomechanical effects of the lumbar posterior complex on the adjacent segments after posterior lumbar interbody fusion (PLIF) surgeries.

**Methods:**

A finite element model of the L1–S1 segment was modified to simulate PLIF with total laminectomy (PLIF-LAM) and PLIF with hemilaminectomy (PLIF-HEMI) procedures. The models were subjected to a 400N follower load with a 7.5-N.m moment of flexion, extension, torsion, and lateral bending. The range of motion (ROM), intradiscal pressure (IDP), and ligament force were compared.

**Results:**

In Flexion, the ROM, IDP and ligament force of posterior longitudinal ligament, intertransverse ligament, and capsular ligament remarkably increased at the proximal adjacent segment in the PLIF-LAM model, and slightly increased in the PLIF-HEMI model. There was almost no difference for the ROM, IDP and ligament force at L5-S1 level between the two PLIF models although the ligament forces of ligamenta flava remarkably increased compared with the intact lumbar spine (INT) model. For the other loading conditions, these two models almost showed no difference in ROM, IDP and ligament force on the adjacent discs.

**Conclusions:**

Preserved posterior complex acts as the posterior tension band during PLIF surgery and results in less ROM, IDP and ligament forces on the proximal adjacent segment in flexion. Preserving the posterior complex during decompression can be effective on preventing adjacent segment degeneration (ASD) following PLIF surgeries.

## Introduction

Posterior lumbar interbody fusion (PLIF) has become a widely accepted surgical procedure to achieve a solid and stable arthrodesis. Nevertheless, adjacent segment degeneration (ASD) has been reported to be a long-term complication after fusion [1, 2]. The occurrence of radiographic ASD (R-ASD) and symptomatic ASD (S-ASD) in lumbar position have been reported to reach 26.6% and 8.5% respectively in long-term follow-ups [1, 2]. It is evident that the number of surgical interventions for ASD will continue to increase as more spinal fusions are performed. Previous biomechanical and prospective clinical studies have demonstrated that important factors leading to adjacent segment motion, stress, and degenerative changes after fusionare construct stiffness, consequences of the fusion itself and consequences of surgical procedures such as radical decompression, which can often be modified at the time of the operation[3, 4].

During PLIF surgeries, it is essential to decompress the involved neural elements by removing all or part of the posterior elements, including the lamina, spinous process, supraspinous ligament (SSL) and interspinous ligament (ISL), ligamentum flavum (LF), and facet joints. In an intact lumbar spine, posterior complex (including spinous process, SSL and ISL) [4] plays a critical role of a tension band mechanism to resist a greater flexed moment [5, 6]. Theoretically, removing the posterior complex during PLIF procedures may influence motion and loadbearing characteristics of the adjacent segment, and thus contributing to the pathogenesis of postoperative instability at the adjacent segment [4, 6, 7].

In fact, several long-term follow-ups of posterolateral fusion with decompressive laminectomy revealed that accelerated degeneration of adjacent segment and segmental instability above the fusion occurred in all their patients [7, 8]. On the other hand, previous studies have reported less ASD after anterior fusion surgeries, which remained the intact posterior structures [9]. A previous study using a posterolateral finite element (FE) model with total laminectomy versus posterolateral fusion with hemilaminectomy, has shown that the SSL and ISL shared some external forces and consequently reduced the stress concentration in adjacent segments, and may contribute to delay ASD [10].

Clinically, previous studies have found that progressive postoperative ASD occurred significantly more frequently in patients conducted by PLIF with total laminectomy [4, 11]. Based on the above results, we hypothesized that preservation of the posterior complex in the decompression procedure has the biomechanical effect in preventing ASD following PLIF surgery. However, no biomechanical study has specifically addressed the effect of preserving posterior elements during PLIF surgeries on postoperative ASD.

In the present study, we developed a FE model to investigate the biomechanical changes resulting from the PLIF models using two comparable decompression techniques, that was, PLIF with LAM (PLIF-LAM) versus PLIF with HEMI (PLIF-HEMI) model, subsequently determined whether preserving the posterior complex had a preventive effect on ASD following PLIF.

## Methods

A previously validated 3-dimensional intact lumbar (INT) FE model (L1–S1) was used [12]. The commercial finite element program package (Abaqus 6.11; Dassault Systèmes Simulia Corporation, France) was used to model the spinal segments. The FE model of the detailed ligamentous lumbar spine included vertebrae, intervertebral discs, facet joints, and the surrounding ligaments: i.e., anterior longitudinal ligament (ALL), posterior longitudinal ligament (PLL), intertransverse ligament (ITL), LF, ISL, SSL, and capsular ligament (CL). The disc annulus consisted of fibers embedded in the ground substance. The spinal vertebrae and intervertebral discs were modeled as 8-node, 3-dimensional solid elements. The annulus ground substance and nucleus pulposus were simulated to be nearly incompressible and hyper-elastic [12, 13]. The spinal ligaments and annulus fibers of discs were modeled as tension-only springs with nonlinear properties taken from the literature [13]. The contact characteristics of the facet articulation were simulated by three-dimensional frictionless contact elements. The gap of the facet joint was < 0.1 mm. The material properties of this FE model were listed in Table 1. To verify the reliability of the intact FE model, the range of motion (ROM) of this INT model was compared with the experimental and simulated data presented by Renner et al [14].

**Table 1:** Material properties used in finite element model of the lumbar spine.

Components	Young's modulus (MPa)	Poisson's ratio	Element type	Element No.
Cortical bone	14000	0.3	Hex	2585
Cancellous bone	100	0.2	Tetra	129931(INT)
				181340(PLIF)
Posterior elements	3500	0.25	Tetra	250978(INT)
				287902(PLIF-HEMI)
				265825(PLIF-LAM)
Endplate	10000	0.25	Hex	4921
Sacrum	5000	0.2	Tetra	200295
Cage	3500	0.3	Tetra	29901
Fixator	110000	0.3	Tetra	39598
Graft	50	0.2	Tetra	43663
Facet cartilage	Neo-Hookean, C_10_ = 2		Hex	7293(INT)
				6654 (PLIF-HEMI)
				6593 (PLIF-LAM)
Annulus	Mooney–Rivlin C_1_ = 0.18, C_2_ = 0.045		Hex	6000 (INT)
				5850 (PLIF)
Nucleus pulposus	Mooney–Rivlin C_1_ = 0.12, C_2_ = 0.03		Hex	7200 (INT)
				5760 (PLIF)
Fiber	Calibrated stress-strain curves		Spring	14400(INT)
				14040 (PLIF)
Ligament	Calibrated deflection-force curves		Spring	234 (INT)
				211 (PLIF-HEMI)
				188 (PLIF-LAM)

INT model: intact lumbar spine; PLIF, posterior lumbar interbody fusion; PLIF-HEMI, posterior lumbar interbody fusion with hemi-laminectomy model; PLIF-LAM, posterior lumbar interbody fusion with total laminectomy model

### PLIF Model

**Fig 1:**
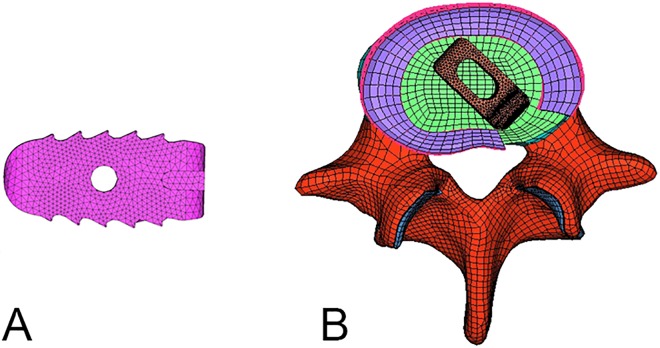
Cage and cross-sectional view of the posterior lumbar interbody fusion model. (A) Lateral view of the polyetheretherketone (PEEK) cage, and (B) cross-sectional view of the posterior lumbar interbody fusion model with one diagonally placed PEEK cage.

In the procedure of the interbody fusion, the entire nucleus, part of the annulus at the posterior-right side were removed. The anterior aspect of the disc space was then firmly packed with cancellous bone graft, followed by asymmetrical placement of a single polyetheretherketone (PEEK) cage (10 × 10 × 22 mm × 4°; B.J.C. capstone; Medtronic Sofamor, Danek, Memphis, TN, USA), diagonally positioned at 45° in the middle and posterior disk space (Fig 1). All the cages were filled with cancellous bone to simulate the embedded bone graft within the implanted cage. The cage-bone interface was modeled by surface-to-surface contact elements to simulate the early postoperative stage after PLIF surgeries. The coefficient of friction at the cage-vertebra interface was 0.2 to mimic small teeth on the contact surfaces [15].

**Fig 2:**
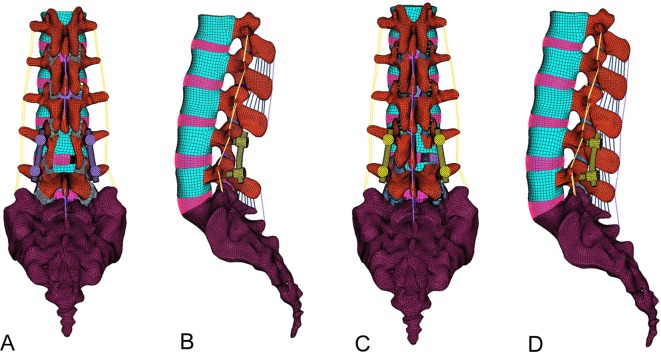
Finite element models of posterior lumbar interbody fusion. (A) Posterior and (B) lateral view of the posterior lumbar interbody fusion with total laminectomy (PLIF-LAM) model, and (C) posterior (D) and lateral view of the PLIF with hemilaminectomy (PLIF-HEMI) model.

The posterior decompressive surgery as total laminectomy includes the resection of L4 posterior midline structures (lamina, spinous process, LF, SSL, ISL, as well as the medial half of the facet joints at the L4–L5 level), so the PLIF-LAM model was constructed (Fig 2A and 2B). The PLIF-HEMI model was also constructed by removing the right lamina, facet joint and LF while preserving the spinous process, contralateral lamina and facet joint, as well as the left LF, SSL and ISL (Fig 2C and 2D). Both PLIF models included spinal fixator made of titanium alloy by using 3-dimensional solid element, so the spinal fixator was through the pedicle and vertebral body. The spinal fixator included four pedicle screws 6.5 mm diameter each, and two rods 5.5 mm diameter.

### Boundary and Loading Conditions

The L1 vertebral body was subjected to a 400-N compressive follower preload [16, 17] while the inferior surface of the S1 vertebra was completely fixed in all degrees of freedom. Moreover, additional 7.5-N.m bending moments were applied to simulate flexion, extension, lateral bending and torsion [18, 19]. Finally, the ROM, the intradiscal pressure (IDP), and the ligament force were computed and compared among the simulated cases.

## Results

### Validation of the Intact FE Model

**Fig 3:**
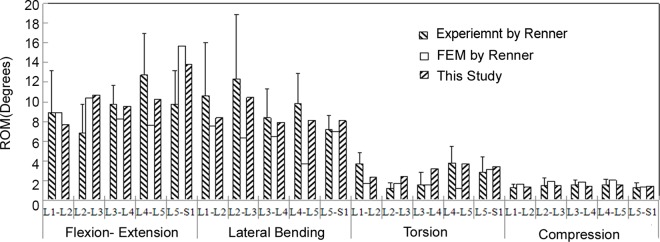
Comparison of Motion between the current intact model and the previous study of Renner et al.

We compared our motion and compression displacement results with those from a previous in vitro study and a FE model conducted by Renner et al. [14] under the same loads to our model. A good agreement was obtained between our results and the previously reported data (Fig 3).

### Range of motion

The ROM at the L4-L5 level of two models was less than that of the intact model in flexion, extension, bending and axial rotation respectively (Table 2). Differences in ROM at the L4–L5 level between two models were less than 0.3° for all loading cases. Both PLIF models had similar stabilization in extension, rotation, and bending cases.

**Table 2:** The ROM among three models in each loading condition.

	Segment	INT	PLIF-HEMI	PLIF-LAM
Flexion	L1-L2	4.94	4.97	4.97
	L2-L3	4.88	4.88	4.91
	L3-L4	5.36	5.59	6.68
	L4-L5	6.15	0.54	0.78
	L5-S1	6.81	6.74	6.80
Extension	L1-L2	3.42	3.56	3.56
	L2-L3	3.35	3.37	3.37
	L3-L4	4.31	4.47	4.53
	L4-L5	5.88	0.14	0.43
	L5-S1	5.04	4.86	5.01
Lateral Bending	L1-L2	4.69	4.76	4.76
	L2-L3	4.91	4.91	4.90
	L3-L4	4.66	4.64	4.64
	L4-L5	4.91	0.59	0.79
	L5-S1	3.55	3.49	3.55
Torsion	L1-L2	2.04	2.06	2.05
	L2-L3	1.89	1.91	1.90
	L3-L4	2.58	2.65	2.68
	L4-L5	2.78	0.52	0.65
	L5-S1	2.64	2.57	2.60

INT model: intact lumbar spine; PLIF-HEMI, posterior lumbar interbody fusion with hemilaminectomy model; PLIF-LAM, posterior lumbar interbody fusion with total laminectomy model

**Fig 4:**
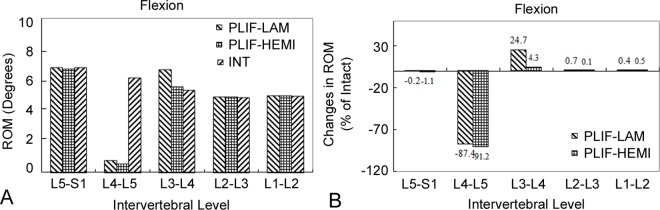
The results of range of motion (ROM) in flexion. (A) Range of motion (ROM) in flexion among the intact (INT) model and the two posterior lumbar interbody fusion (PLIF) models, and (B) percentage change in ROM between the two PLIF models during flexion. Percentage change = (Data of surgical model—Data of intact model)/Data of intact model ×100%.

Compared with the INT model, the ROM of the PLIF-LAM and PLIF-HEMI models respectively increased by 24.7% and 4.3% at the L3–L4 level during flexion (Fig 4). For the other loading conditions, these two models had almost no difference in ROM on the adjacent discs.

### Intradiscal Pressure of the Adjacent Segments

**Fig 5:**
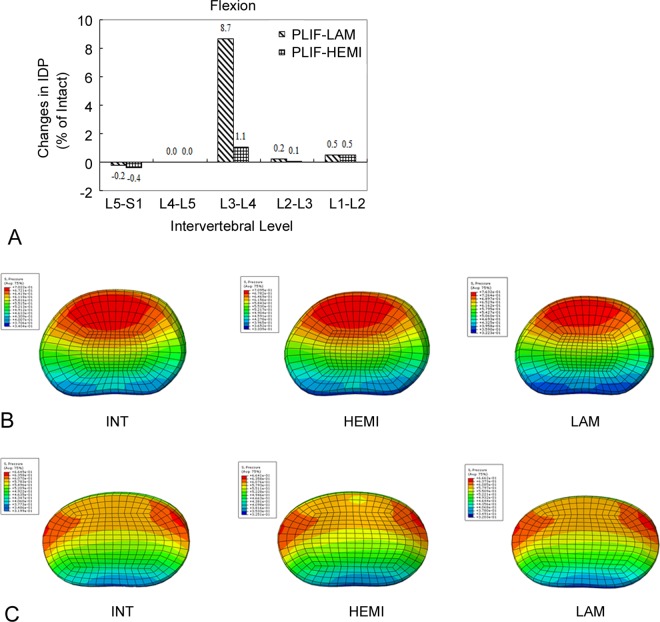
The results of intradiscal pressure (IDP) in flexion. Percentage change of IDPs in flexion between the two PLIF models (A). Percentage change = (Data of surgical model - Data of intact model)/Data of intact model ×100%. Contour plots of IDP at (B) L3-L4 and (C) L5-S1 levels.

The only difference in IDP was found during flexion at the L3–L4 level: the IDP increased by 8.7% in the PLIF-LAM model and 1.1% in the PLIF-HEMI model respectively as compared with that of the INT model, while differences in IDP for the other loading conditions were less than 1.2% (Fig 5). In contrast, the IDPs of the PLIF-HEMI model were similar to those of the INT model for all adjacent levels.

### Ligament Forces

Compared with the INT model, the forces of the preserved ligaments remarkably decreased at the fused segment in the two PLIF models, owing to a remarkable decrease in ROM at the fused level. These values were not different between these two models.

**Fig 6:**
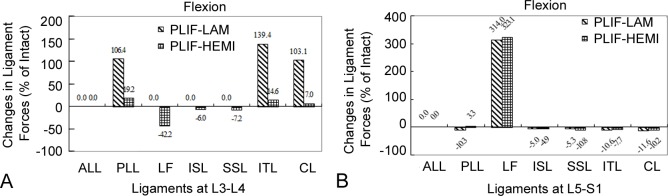
Results of the ligament forces at the L3-L4 and L5-S1 levels in flexion. Percentage change in the ligament forces at the L3-L4 (A) and L5-S1 (B) levels in flexion. Percentage change = (Data of surgical model—Data of intact model)/Data of intact model ×100%.

In flexion, the force of the PLL, ITL and CL at the L3-L4 level increased by 19.2%, 14.6% and 7.0% in the PLIF-HEMI model, and by 106.4%, 139.4% and 103.1% in the PLIF-LAM model respectively. In the PLIF-LAM model, the SSL, ISL and LF at the L3-L4 level had no tensile force for all loading conditions since the laminectomy surgery removed the anchoring point of the three ligaments. In the PLIF-HEMI model, the force of ISL and SSL respectively decreased by 6.0% and 7.2%. The force of the LF at the L3-L4 level decreased by 42.2% since the hemilamintectomy surgery removed the anchoring point of the LF on the right side (Fig 6A).

Compared with the INT model, the ligament force of the LF at the L5/S1 level respectively increased by 323.1% and 314.0% for the PLIF-LAM and PLIF-HEMI models (Fig 6B). For the other loading conditions, the ligament forces in the two PLIF models were similar to those in the model.

## Discussion

Decompression is a routine procedure during PLIF surgery, involving the removal of all or part of the posterior elements. Posterior complex including spinous process, SSL and ISL is the main stabilizing elements as the lumbar spine is flexed within the physiological motions [3, 4]. From the biomechanical point of view, the removal of the posterior complex eliminates the tension band effect in the flexion motion and causes the accelerated development of ASD [7, 8, 20]. The present simulation study was to investigate the biomechanical effect of the posterior complex on adjacent segments by comparing total laminectomy PLIF and hemilaminectomy PLIF model techniques by using finite element analysis.

Our study indicated that the PLIF-LAM and PLIF-HEMI models were able to acquire similar stabilization through the construction of spinal fixators and bone graft, even if the lamina and posterior ligaments were removed. The FE model results showed that the increases in the ROM and IDP were found at the proximal adjacent segment of the PLIF-LAM model. These great changes in the ROM and IDP could be used to interpret the clinical findings of early degeneration of adjacent segment following PLIF surgeries [11, 21, 22].

The greatest increase in ROM and IDP occurred for the flexion loading condition at the L3–L4 level. Indeed, compared with the INT model, the ROM at the proximal adjacent level increased by 24.7% for the PLIF-LAM model and by 4.3% for the PLIF-HEMI model in flexion. This ROM increase was accompanied by an increase in IDP. The IDP of the PLIF-LAM model increased by 8.7% while only a slight increase (1.1%) was found in PLIF-HEMI model as compared with the INT model. Our results suggest that the patients who underwent posterior lumbar interbody fusion surgery with total laminectomy operation are likely to experience a higher incidence of ASD than those who underwent posterior lumbar interbody fusion with hemi-laminectomy operations, which were in accordance with the previous clinical studies [3, 11]. Biomechanically, the SSL and ISL act as a tension band in an intact spine, especially during flexion [23, 24]. In the PLIF-LAM model, the removal of the whole L4 spinous process would damage the SSL, ISL or the anchoring point of the neighboring unfused segments, jeopardize the effect of the tension band, and thus causing the accelerated development of ASD. In contrast, in the PLIF-HEMI model, the preserved posterior complex was able to share external forces and consequently alleviated the IDP in the adjacent discs. On the other hand, Sim et al. [16] found the similar biomechanical properties regarding ROM and IDP at the adjacent segments for the PLIF and transforaminal lumbar interbody fusion (TLIF) groups when using human L2-S2 cadaveric spine specimens, which was probably due to the fact that the posterior complex was kept intact in both groups. Besides, these values were not statistically increased from those of the intact spine [16]. Clinically, the preservation of the posterior complex also allows resuture the lumbar dorsal fascia to the SSL and ISL [4, 7, 10]. These structures provide important bony attachments for the posterior stabilizing paraspinal muscles acting as active spinal stabilizers. Our findings supported the clinical observations that preservation of the posterior complex in fused segment decompression should result in better protection against ASD [4, 11].

In flexion, the changes in distribution of ligament forces at the proximal adjacent segment level were different between the two PLIF models. The ligament forces of the PLL, ITL and CL remarkably increased at the proximal adjacent segment in the PLIF-LAM model with the removal of the posterior complex, whereas the forces of the PLL, ITL and FC slightly increased in the PLIF-HEMI model with the preserved posterior complex. Biomechanically, the preserved posterior complex in the PLIF-HEMI model was able to share external forces, which is the dominant component of the resistance to flexion in an intact spine [10]. Oppositely, the removal of the posterior complex eliminated the tension band effect in the flexion motion [4, 7], which may thus produce larger forces on the PLL, ITL and CL to resist to flexion loading. Several authors reported that the changes in load sharing among ligaments would alter the normal physiological and mechanical environments of ligaments, leading to ligament failure and hypertrophy [24, 25]. Moreover, the increase in ligaments forces were likely relevant to the invocation of pain and prone to cause chronic soft tissue injury, facet joint degeneration as well as hypertrophy of the LF, and thereby cause the ASD [5, 23, 24, 26].

On the other hand, there was almost no difference in ligament forces at the L5–S1 level between the two PLIF models. Interestingly, although the posterior complex was kept intact at the distal adjacent level in the two surgical models, there was a marked increase in ligament forces on LFs at the L5–S1 level comparing with the INT model. Our results also indicated that the loading of ligaments at the dorsal adjacent segment may be changed by the altered stress caused by the rigid instrumentations and fusion, regardless of the preservation or removal of the posterior complex between the fused and adjacent segments. Recently, non-fusion technologies such as posterior dynamic stabilization (PDS) devices can restore the stability of the lumbar spine without adverse stress-shielding effects [27]. Further study would investigate whether the introduction of PDS induces a substantial reduction in the ligament force rise at the dorsal adjacent level.

Compared with previous PLIF FE models [16, 17, 24], the current PLIF FE model focused on the important role of posterior complex. Biomechanically, posterior complex acts as the posterior tension band in flexion. A previous FE analysis has shown that removing the posterior complex during decompression with posterolateral fusion could cause potential adjacent segmental instability [10]. Moreover, PLIF has been reported to be more rigid than posterolateral fusion [28, 29], and hence patients who undergo are likely to experience a higher incidence of ASD than those who underwent posterolateral fusion [30]. However, most previous PLIF FE analyses have neglected the importance of posterior complex and focused on the altered biomechanical behavior in the fused lumbar spine with spinal fixators and cages [16, 17, 24, 28, 29]. Therefore, our FE model found that the preserved posterior complex led to less ROM, IDP and ligament forces on the proximal adjacent segment in flexion.

Our data were based upon FE analysis and hence had several limitations. Firstly, the material properties of simulation are slightly simplified and idealized. More accurate geometrical and material properties should be considered in future work. Secondly, only relative values of IDP and ligament force were used and compared since the two parameters were difficult to obtain from cadaveric experiments. Thirdly, the muscle contractions may bring complicated external forces that could have significant influences on the biomechanical perspective. Finally, we only performed L4–L5 level fusion in our biomechanical study. However, results may vary if the fusion is performed in other segments, or in two-level fusion. However, although some aspects were simplified in our FE model, it was well validated by the previous in vitro study. Therefore, the model established in this study is valid and can be used as an efficient tool to evaluate the effects of the two surgical scenarios on the lumbar spine.

## Conclusion

The preserved posterior complex acts as the posterior tension band during PLIF surgery and results in less ROM, IDP and ligament forces on the proximal adjacent segment in flexion. Therefore, preserving the posterior complex during decompression can be effective in preventing ASD following PLIF surgeries.

## Supporting Information

S1 Fig Cage and cross-sectional view of the posterior lumbar interbody fusion model. (A) Lateral view of the polyetheretherketone (PEEK) cage, and (B) cross-sectional view of the posterior lumbar interbody fusion model with one diagonally placed PEEK cage.
(TIF)

S2 Fig Finite element models of posterior lumbar interbody fusion. (A) Posterior and (B) lateral view of the posterior lumbar interbody fusion with total laminectomy (PLIF-LAM) model, and (C) posterior (D) and lateral view of the PLIF with hemilaminectomy (PLIF-HEMI) model.
(TIF)

S3 Fig Comparison of Motion between the current intact model and the previous study of Renner et al. (TIF)

S4 Fig The results of range of motion (ROM) in flexion. (A) Range of motion (ROM) in flexion among the intact (INT) model and the two posterior lumbar interbody fusion (PLIF) models, and (B) percentage change in ROM between the two PLIF models during flexion. Percentage change = (Data of surgical model—Data of intact model)/Data of intact model ×100%.
(TIF)

S5 Fig The results of intradiscal pressure (IDP) in flexion. Percentage change of IDPs in flexion between the two PLIF models. Percentage change = (Data of surgical model—Data of intact model)/Data of intact model ×100%. Contour plots of IDP at (C) L3-L4 and (D) L5-S1 levels.
(TIF)

S6 Fig Results of the ligament forces at the L3-L4 and L5-S1 levels in flexion. Percentage change in the ligament forces at the L3-L4 (A) and L5-S1 (B) levels in flexion. Percentage change = (Data of surgical model—Data of intact model)/Data of intact model ×100%.
(TIF)

S1 Table Material properties used in finite element model of the lumbar spine. INT model: intact lumbar spine; PLIF, posterior lumbar interbody fusion; PLIF-HEMI, posterior lumbar interbody fusion with hemi-laminectomy model; PLIF-LAM, posterior lumbar interbody fusion with total laminectomy model.
(PDF)

S2 Table The ROM among three models in each loading condition. INT model: intact lumbar spine; PLIF-HEMI, posterior lumbar interbody fusion with hemilaminectomy model; PLIF-LAM, posterior lumbar interbody fusion with total laminectomy model.
(PDF)
