# Correction: Preserving Posterior Complex Can Prevent Adjacent Segment Disease following Posterior Lumbar Interbody Fusion Surgeries: A Finite Element Analysis

**DOI:** 10.1371/journal.pone.0172329

**Published:** 2017-02-13

**Authors:** Yun-Peng Huang, Cheng-Fei Du, Cheng-Kung Cheng, Zheng-Cheng Zhong, Xuan-Wei Chen, Gui Wu, Zhe-Chen Li, Jin-Duo Ye, Jian-Hua Lin, Li Zhen Wang

The seventh author's name is spelled incorrectly. The correct name is: Zhe-Chen Li.
