# Supplementary figures and images for: Preserving Posterior Complex Can Prevent Adjacent Segment Disease following Posterior Lumbar Interbody Fusion Surgeries: A Finite Element Analysis
Source: PLoS One. 2016 Nov 21;11(11):e0166452. doi: 10.1371/journal.pone.0166452 (PMC5117648; doi:10.1371/journal.pone.0166452)

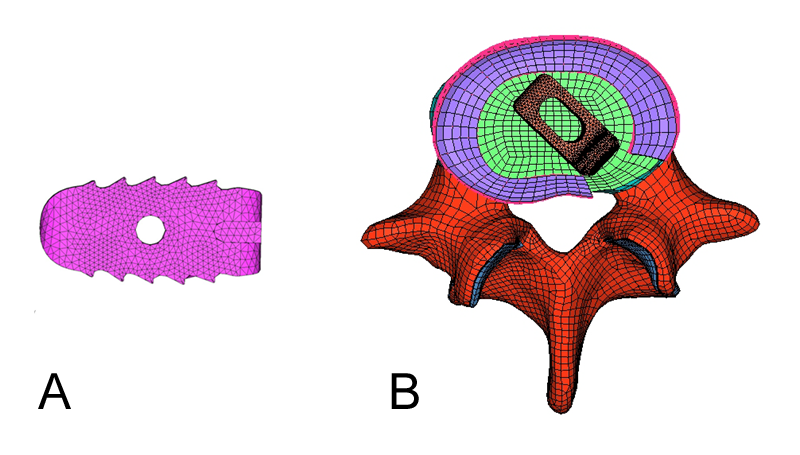

Supplement: S1 Fig — (A) Lateral view of the polyetheretherketone (PEEK) cage, and (B) cross-sectional view of the posterior lumbar interbody fusion model with one diagonally placed PEEK cage. (TIF) [file pone.0166452.s001.tif]

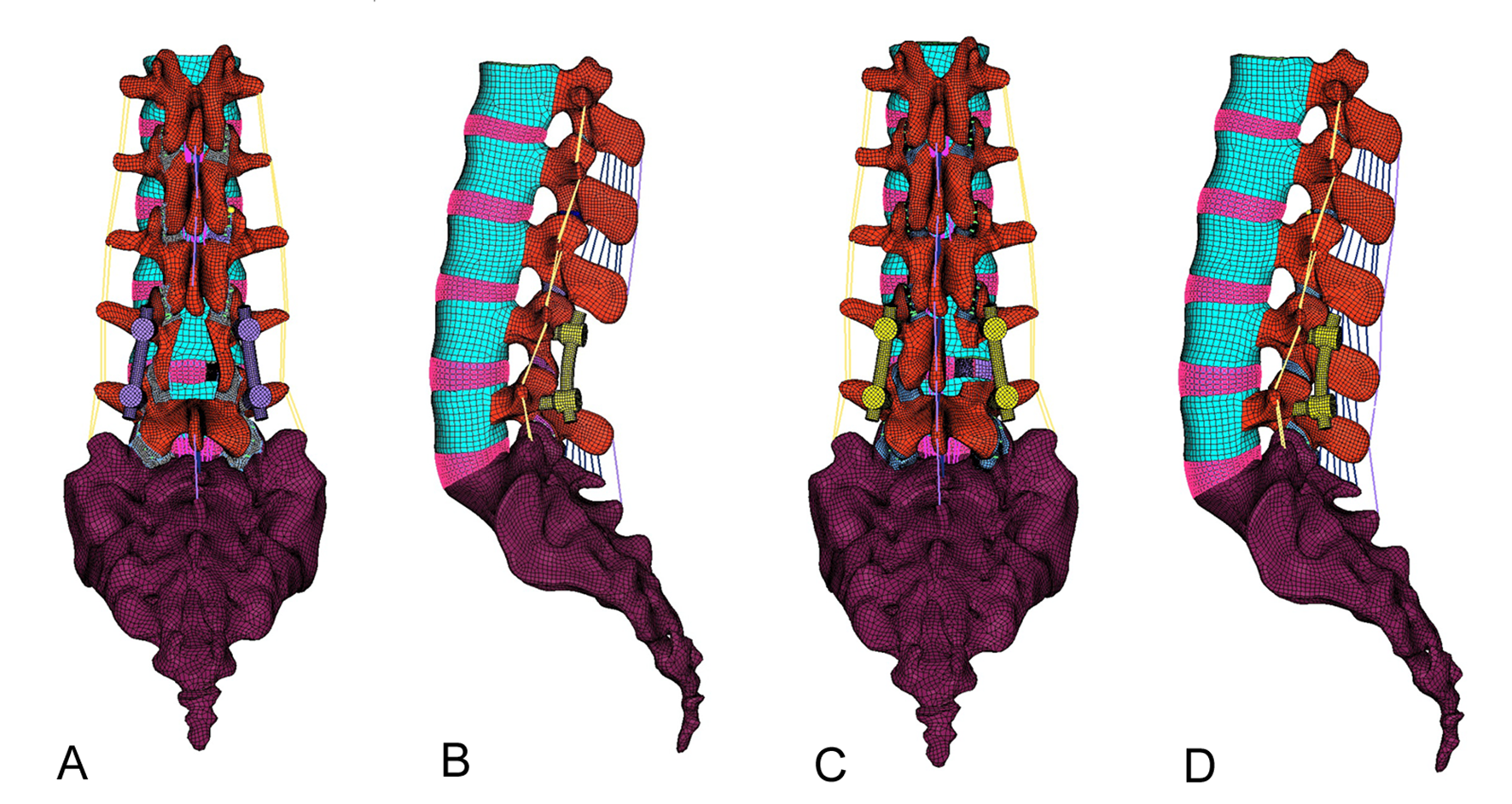

Supplement: S2 Fig — (A) Posterior and (B) lateral view of the posterior lumbar interbody fusion with total laminectomy (PLIF-LAM) model, and (C) posterior (D) and lateral view of the PLIF with hemilaminectomy (PLIF-HEMI) model. (TIF) [file pone.0166452.s002.tif]

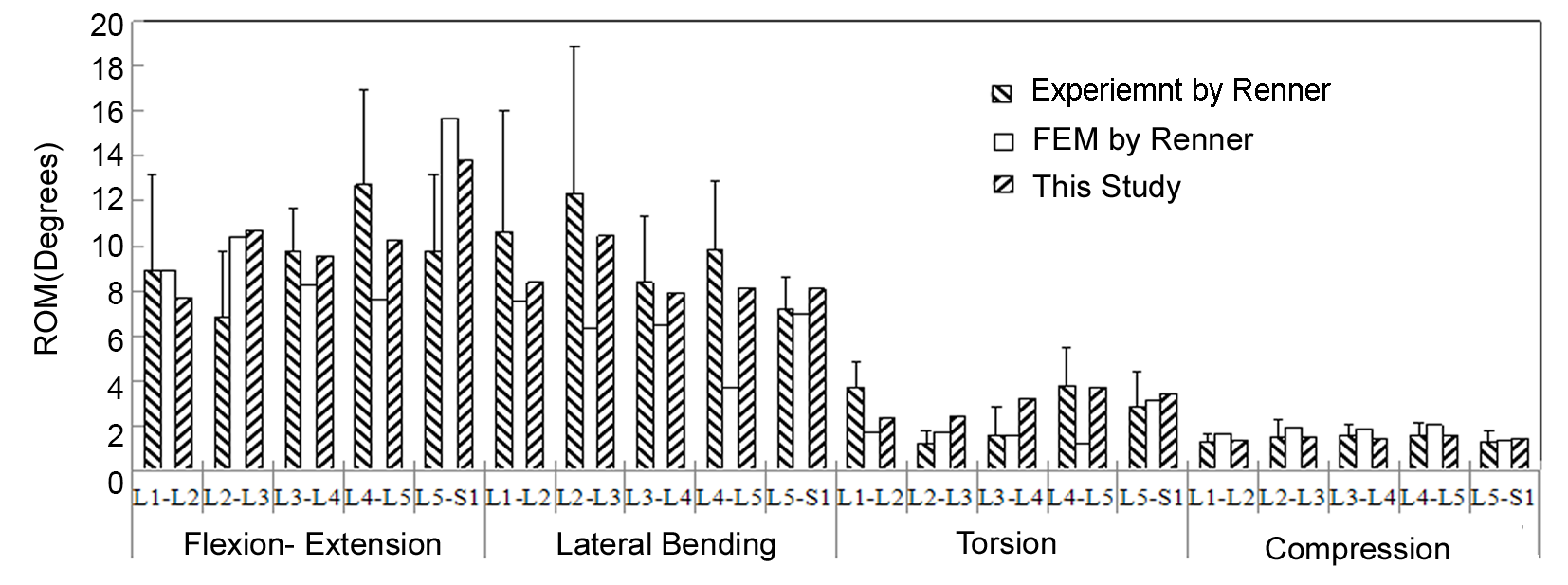

Supplement: S3 Fig — (TIF) [file pone.0166452.s003.tif]

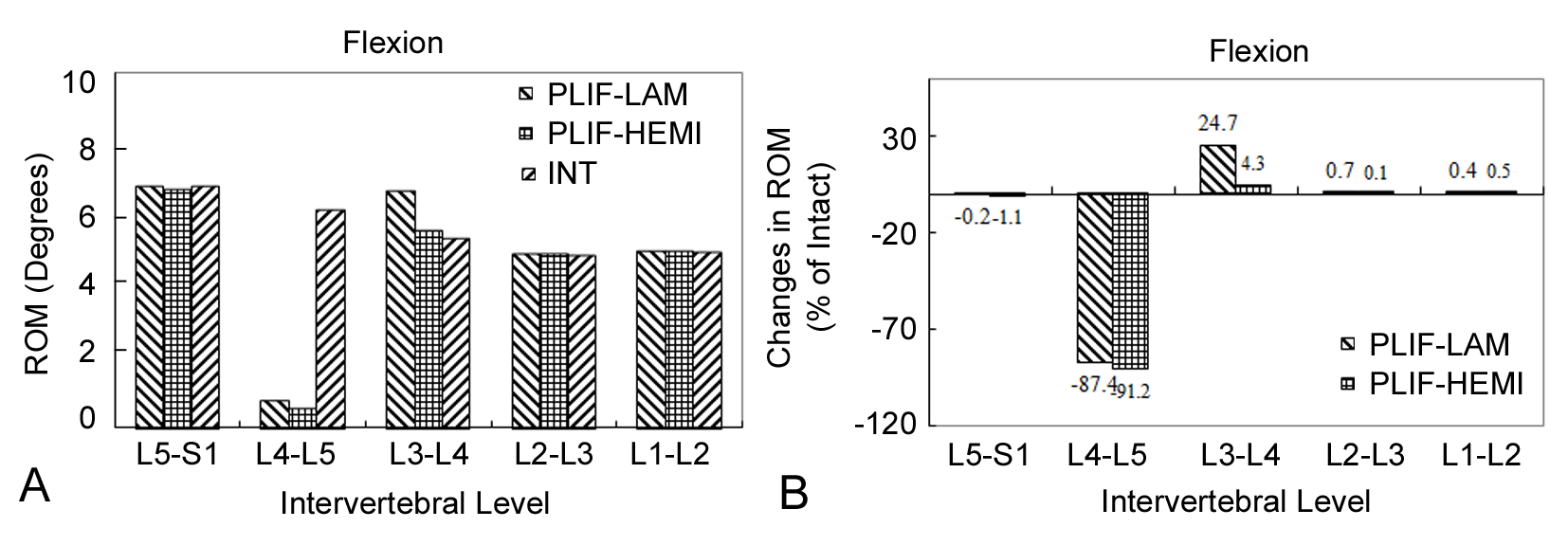

Supplement: S4 Fig — (A) Range of motion (ROM) in flexion among the intact (INT) model and the two posterior lumbar interbody fusion (PLIF) models, and (B) percentage change in ROM between the two PLIF models during flexion. Percentage change = (Data of surgical model—Data of intact model)/Data of intact model ×100%. (TIF) [file pone.0166452.s004.tif]

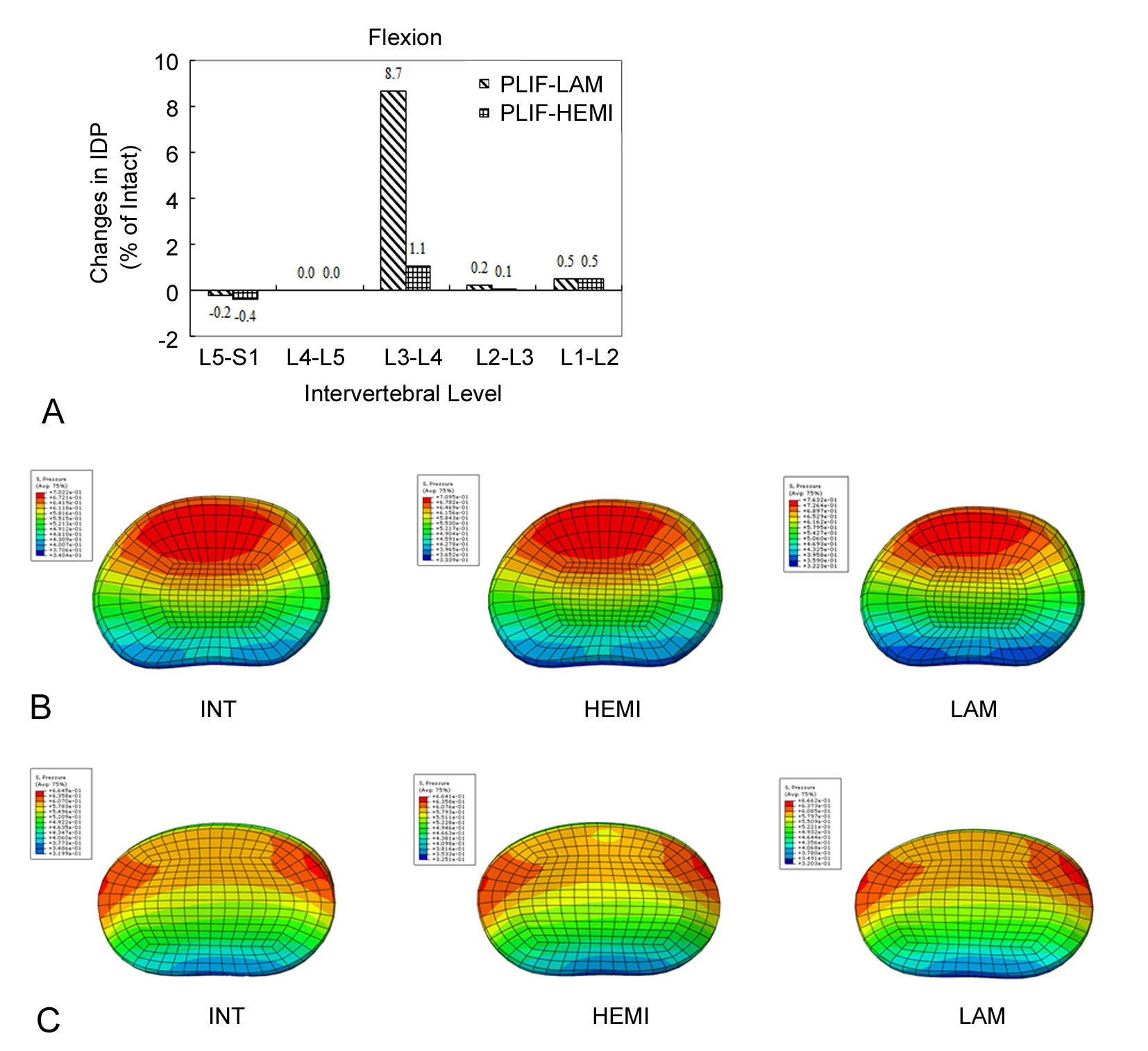

Supplement: S5 Fig — Percentage change of IDPs in flexion between the two PLIF models. Percentage change = (Data of surgical model—Data of intact model)/Data of intact model ×100%. Contour plots of IDP at (C) L3-L4 and (D) L5-S1 levels. (TIF) [file pone.0166452.s005.tif]

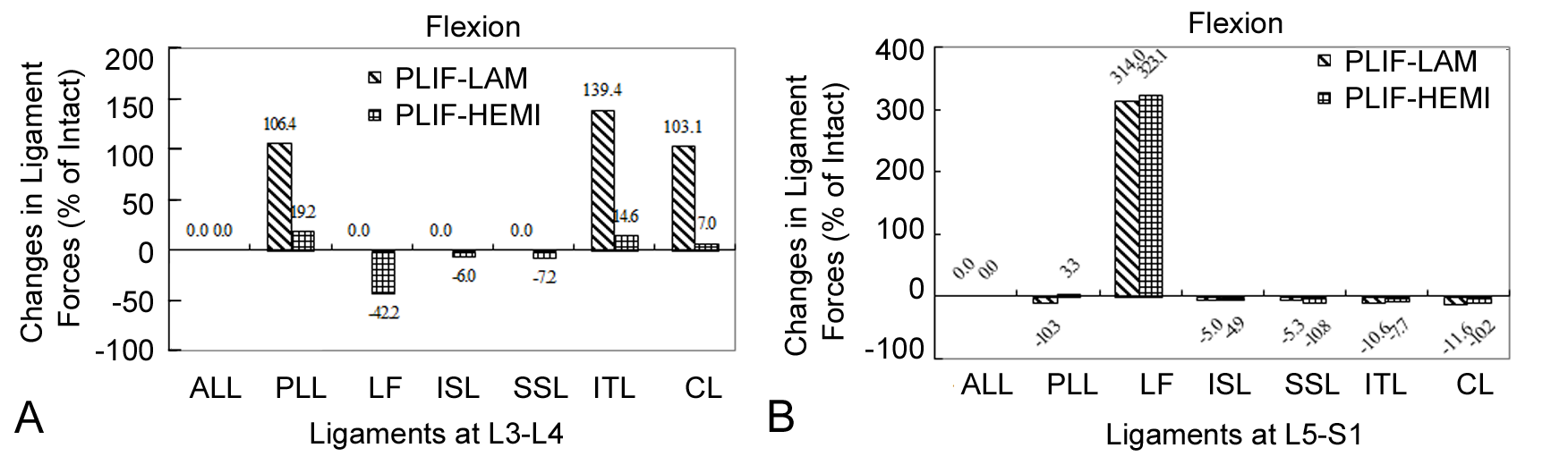

Supplement: S6 Fig — Percentage change in the ligament forces at the L3-L4 (A) and L5-S1 (B) levels in flexion. Percentage change = (Data of surgical model—Data of intact model)/Data of intact model ×100%. (TIF) [file pone.0166452.s006.tif]
