# Supplementary material for: Preserving Posterior Complex Can Prevent Adjacent Segment Disease following Posterior Lumbar Interbody Fusion Surgeries: A Finite Element Analysis
Source: PLoS One. 2016 Nov 21;11(11):e0166452. doi: 10.1371/journal.pone.0166452 (PMC5117648; doi:10.1371/journal.pone.0166452)
Supplement: S1 Table — INT model: intact lumbar spine; PLIF, posterior lumbar interbody fusion; PLIF-HEMI, posterior lumbar interbody fusion with hemi-laminectomy model; PLIF-LAM, posterior lumbar interbody fusion with total laminectomy model. (PDF) [file pone.0166452.s007.pdf]

Table 1 Material properties used in finite element model of the lumbar spine

Components	Young's modulus (MPa)	Poisson's ratio	Element type	Element No.
Cortical bone	14000	0.30	Hex	2585
Cancellous bone	100	0.2	Tetra	129931(INT)<br>181340(PLIF)
Posterior elements	3500	0.25	Tetra	250978(INT)<br>287902(PLIF-HEMI)<br>265825(PLIF-LAM)
Endplate	10000	0.25	Hex	4921
Sacrum	5000	0.2	Tetra	200295
Cage	3500	0.3	Tetra	29901
Fixator	110000	0.3	Tetra	39598
Graft	50	0.2	Tetra	43663
Facet cartilage	Neo-Hookean, C_{10}=2		Hex	7293(INT)<br>6654(PLIF-HEMI)<br>6593(PLIF-LAM)
Annulus	Mooney–Rivlin C_1 = 0.18, C_2 = 0.045		Hex	6000(INT)<br>5850(PLIF)
Nucleus pulposus	Mooney–Rivlin C_1 = 0.12, C_2 = 0.03		Hex	7200(INT)<br>5760(PLIF)
Fiber	Calibrated curves	stress -strain	Spring	14400(INT)<br>14040(PLIF)
Ligament	Calibrated curves	deflection -force	Spring	234(INT)<br>211(PLIF-HEMI)<br>188(PLIF-LAM)

INT model: intact lumbar spine; PLIF, posterior lumbar interbody fusion; PLIF-HEMI, posterior lumbar interbody fusion with hemi-laminectomy model; PLIF-LAM, posterior lumbar interbody fusion with total laminectomy model;
