# Supplementary material for: Preserving Posterior Complex Can Prevent Adjacent Segment Disease following Posterior Lumbar Interbody Fusion Surgeries: A Finite Element Analysis
Source: PLoS One. 2016 Nov 21;11(11):e0166452. doi: 10.1371/journal.pone.0166452 (PMC5117648; doi:10.1371/journal.pone.0166452)
Supplement: S2 Table — INT model: intact lumbar spine; PLIF-HEMI, posterior lumbar interbody fusion with hemilaminectomy model; PLIF-LAM, posterior lumbar interbody fusion with total laminectomy model. (PDF) [file pone.0166452.s008.pdf]

Table 2 The ROM among three models in each loading condition

	Segment	INT	PLIF-HEMI	PLIF-LAM
Flexion	L1-L2	4.94	4.97	4.97
	L2-L3	4.88	4.88	4.91
	L3-L4	5.36	5.59	6.68
	L4-L5	6.15	0.54	0.78
	L5-S1	6.81	6.74	6.80
Extension	L1-L2	3.42	3.56	3.56
	L2-L3	3.35	3.37	3.37
	L3-L4	4.31	4.47	4.53
	L4-L5	5.88	0.14	0.43
	L5-S1	5.04	4.86	5.01
Lateral Bending	L1-L2	4.69	4.76	4.76
	L2-L3	4.91	4.91	4.90
	L3-L4	4.66	4.64	4.64
	L4-L5	4.91	0.59	0.79
	L5-S1	3.55	3.49	3.55
Torsion	L1-L2	2.04	2.06	2.05
	L2-L3	1.89	1.91	1.90
	L3-L4	2.58	2.65	2.68
	L4-L5	2.78	0.52	0.65
	L5-S1	2.64	2.57	2.60

INT model: intact lumbar spine; PLIF-HEMI, posterior lumbar interbody fusion with hemilaminectomy model; PLIF-LAM, posterior lumbar interbody fusion with total laminectomy model;
